# Impact of Individual Headache Types on the Work and Work Efficiency of Headache Sufferers

**DOI:** 10.3390/ijerph17186918

**Published:** 2020-09-22

**Authors:** Svetlana Simić, Tamara Rabi-Žikić, José R. Villar, José Luis Calvo-Rolle, Dragan Simić, Svetislav D. Simić

**Affiliations:** 1 Faculty of Medicine, University of Novi Sad, 21000 Novi Sad, Serbia; svetlana.simic@mf.uns.ac.rs (S.S.); tamara.rabi-zikic@mf.uns.ac.rs (T.R.-Z.); 2 Faculty of Geology, Campus de Llamaquique, University of Oviedo, 33005 Oviedo, Spain; villarjose@uniovi.es; 3 Department of Industrial Engineering, University of A Coruña, 15405 Ferrol-A Coruña, Spain; jlcalvo@udc.es; 4 Faculty of Technical Sciences, University of Novi Sad, 21000 Novi Sad, Serbia; simicsvetislav@uns.ac.rs

**Keywords:** working population, headache burden, migraine, tension-type headache

## Abstract

*Background:* Headaches have not only medical but also great socioeconomic significance, therefore, it is necessary to evaluate the overall impact of headaches on a patient’s life, including their work and work efficiency. The aim of this study was to determine the impact of individual headache types on work and work efficiency. *Methods*: This research was designed as a cross-sectional study performed by administering a questionnaire among employees. The questionnaire consisted of general questions, questions about headache features, and questions about the impact of headaches on work. *Results*: Monthly absence from work was mostly represented by migraine sufferers (7.1%), significantly more than with sufferers with tension-type headaches (2.23%; *p* = 0.019) and other headache types (2.15%; *p* = 0.025). Migraine sufferers (30.2%) worked in spite of a headache for more than 25 h, which was more frequent than with sufferers from tension-type and other-type headaches (13.4%). On average, headache sufferers reported work efficiency ranging from 66% to 90%. With regard to individual headache types, this range was significantly more frequent in subjects with tension-type headaches, whereas 91–100% efficiency was significantly more frequent in subjects with other headache types. Lower efficiency, i.e., 0–40% and 41–65%, was significantly more frequent with migraine sufferers. *Conclusions*: Headaches, especially migraines, significantly affect the work and work efficiency of headache sufferers by reducing their productivity. Loss is greater due to reduced efficiency than due to absenteeism.

## 1. Introduction

Headaches are one of the most frequent complaints in medicine in general and a frequent disorder in the working population that significantly affects absenteeism and loss of productivity; however, its effect on work efficiency has been insufficiently studied [1,2,3]. Some primary headaches are commonly linked with a broad range of psychiatric comorbidities [4]. Considering that the working population is the mainstay of society, the burden of an employee’s illness cannot only be viewed through the costs of diagnosis and treatment, but also through loss incurred due to absence from work and reduced productivity [5,6]. Health economists in the field of headaches deal with measuring the economic burden of headaches, i.e., the cost of headaches. The cost of illness is assessed through direct, indirect, and non-material costs. Direct costs are expenses made through evaluation and treatment, indirect costs are assessed through lost productivity [6,7], and nonmaterial costs occur due to reduced quality of life. The costs of pain and suffering are difficult to measure, and this aspect is therefore frequently neglected. However, the greatest economic burden of migraines mostly lies in the realm of indirect costs [7,8,9].

Economics deals with the ways a society allocates its resources to possible ends. Health economics is a branch that has its application in the health-care system [10]. It has its limitations and specificities. A practicing physician faces an almost impossible task of being rational and economical while using the most recent approaches to diagnose and treat their patients. In less developed countries, this problem is even greater [11]. There is a strong association between the economy and healthcare of a country, and it needs to be dealt with thoroughly, especially when talking about the health of the working population [12]. A population is expected to give a lot in terms of the production of goods and services and is generally considered healthy. As such, it is frequently (un)consciously neglected. In transitional countries, the health of the working population, which is the mainstay of transitional changes, is often marginalized. The same occurs with people suffering from primary headaches.

Studies on the association of different diseases, including headaches, with the development of disability, and the effects of illness on absenteeism and reduced productivity were carried out mostly in developed countries [13,14,15]. For example, studies found that musculoskeletal disorders not only impair the health status of the professional population, but also impact their ability to work [16]. How certain therapeutic procedures can contribute to decreased productivity loss has also been studied. It was shown that productivity loss is less when early migraine treatment is applied, and when pain is not moderate to severe, either with triptan or non-triptan treatment [11,13]. Migraine patients treated with triptans showed that those with an insufficient response to triptan medication have significantly worse health-related quality of life and are less productive than those who respond to triptans [17]. Furthermore, pharmacoeconomic research demonstrated that early interventions with triptans have more benefits than delayed interventions do [13,14]. The education of both health professionals and patients is alsosignificant. Better education means better treatment results and less economic loss [18,19]. Such studies were mostly carried out on a national level in developed countries. However, some less developed countries also conducted studies on headaches [20]. In Serbia, no such research has been carried out, and when the prevalence or economic burden of a disease need to be evaluated, approximations of published data are often used. Our research is, therefore, intended as contribution to wider insight into the real picture of the impact of headaches on work and work efficiency in the working population in our environment. In this study, we aimed to determine the impact of individual headache types on work and work efficiency.

## 2. Materials and Methods

The study was completed on a sample of 1022 employed subjects, who filled in the questionnaire (see Questionnaire S1) in their workplace. Results presented in the present study are part of a larger study on headaches in the working population of Novi Sad (Serbia). The research was approved by the Ethics Committee of the Clinical Center of Vojvodina on 4 June 2020 (no. 00-215). The management of companies included in the study approved the survey, and all subjects received written information about the research and signed an informed-consent form.

The following questionnaires were used: (1) general questionnaire to select employees suffering from headaches; (2) questionnaire about the characteristics of headaches based on the International Classification of Headache Disorders (ICHD) criteria for headache types [21,22]; and (3) questionnaire about the impact of headaches on life and work. In the present study, we used only the part that related to the effects of headaches on work; this part of the questionnaire contained questions related to work absence, working with a headache, efficiency when working with a headache, and ways in which headaches affect work, e.g., whether the employee worked slower and/or with reduced attention and precision. 

Statistical analysis was undertaken using Statistical Package for Social Sciences version 17.0 (IBM Corp., Armonk, NY, USA). Descriptive statistics was used to describe variables in the research; *p* < 0.05 was considered as statistically significant. We used a contingency table for examining the relationships between categorical variables. Data were analyzed using a chi-squared homogeneity test. A coefficient of determination was used to explain how much the variability of one factor could have been caused by its relationship to another factor.

## 3. Results

Results presented in this paper show that, out of the 1022 subjects who completed the survey, 579 (56.65%) reported headaches; 169 (16.53%) had migraines, 224 (21.91%) had tension-type headaches (TTHs), and 186 (18.19%) had other headache types. The category of other headache types included patients with a headache that, according to their answers, could not be classified as a migraine or tension-type headache. Only subjects with headaches were further analyzed.

As shown in Table 1, work absenteeism due to headaches was not significantly present. Out of 579 respondents, only 21 (3.63%) had been absent from work in the previous month, which was not statistically significant (*p* = 0.000). Work absenteeism was most prevalent with migraine sufferers. “Yes” to the question of absence from work during the previous month was mostly represented by patients with migraines (7.1%), significantly more than with patients with tension-type headaches (2.23%; *p* = 0.019) and other headache types (2.15%; *p* = 0.025).

Table 2 shows that migraine sufferers spent most hours in a month working in spite of a headache. Namely, 30.2% of migraine sufferers spent more than 25 h working with a headache. Subjects with tension-type headache most frequently (39.7%) worked with headache for 9–24 h in a month. Subjects with other headache types most frequently worked in spite of a headache (49.5%) for 1–8 h in a month. In fact, during the month, employees suffering from migraine headaches often had more than 3 working days, those suffering from tension-type headaches had 1–3 working days, and employees suffering from other headache types had 1 working day per month.

As demonstrated in Table 3, three headache types differed most with regard to hours spent working with a headache and with regard to efficiency while working with a headache, whereas they differed the least with regard to absence from work in the past month. The least efficient were subjects with migraines.

All studied headache-group subjects, as represented in Table 4, most frequently worked with an efficiency of 66–90%, which was statistically significantly higher in tension-type headaches. Higher efficiency was found in subjects with other headache types. The least efficient were subjects with migraines.

According to the International Classification of Headache Disorders (ICHD) criteria [17,18], migraines are divided into migraines without aura (MWoA) and migraines with aura (MWA). Our study shows that, in the group of migraine patients, there were 103 (61%) MWoA and 66 (39%) MWA subjects. There were three subjects in the MWA patient group and two subjects in the MWoA patient group with chronic migraines. Unfortunately, this is a small sample for reliable analysis. Tension-type headaches (TTHs) are divided into infrequent episodic tension-type headaches (infrequent TTHs), frequent episodic tension-type headaches (frequent TTHs), and chronic tension-type headaches (chronic TTHs). Among TTH patients in our sample, there were 116 (52%) with infrequent episodic tension-type headaches, 99 (44%) patients had frequent episodic tension-type headaches, and 9 (4%) patients had chronic tension-type headaches. On the basis of subject responses about the impact of headaches on work, the characteristics of each headache subtype were determined and they are presented in Table 5. The character of each subsample of headache types was most defined by working in spite of a headache, since the contribution of the item to the characteristics was 51.93%, followed by efficiency while working with a headache (19.29%), absence from work (12.76%), effort (6.53%), attention and precision (6.23%), and absence from work in the past month (3.26%).

Subjects with migraines with aura (MWA) and subjects with chronic tension-type headaches spent the greatest amount of time working with a headache. Subjects with migraines with aura were also the least efficient when working with a headache.

## 4. Discussion

In our cultural environment, there is a widespread model of behavior that is little related to individual social and educational status. The patient and their family approach the doctor before any procedures even begin and, without asking, they state that they would do anything necessary and buy all required medications, no matter whether they are covered by health insurance because “good health is invaluable“. However, if initial studies and treatment do not produce satisfactory results in terms of a clear diagnosis and a favorable treatment outcome, almost all of them try to fit in healthcare coverage that gives them financial benefits, namely, the cost of things is an inevitable fact of life.

The impact of headache disorders is a problem of enormous proportions, both for individuals and society [23,24]. The cost–benefit and risk–benefit ratios are two of the most relevant items in ongoing health-organization procedures [25]. Physicians frequently find themselves in situations where they have to consider the economic aspects of a treatment even though they have not been trained for it. Healthcare requires making decisions about diagnosis and treatment that are compatible with current medical opinions along with taking care of its availability and patient compliance to treatment [11,14]. Otherwise, a medical examination is only one more expense. Therefore, designing a health policy involves economists who may facilitate hard decisions that could be inevitable in healthcare, and offer a framework where the costs and benefits of competitive interventions may be compared in an explicit way [8,11].

Headaches are a common complaint in the working population. It is diagnosed according to the ICHD criteria [21,22], and we complied with these criteria in our research. Headaches are the most frequent global health problem, occurring in all age groups, with primary headaches affecting more than half of the adult population [26]. Chronic migraines with low prevalence produce an extremely high disability [27]. The peak of prevalence of the most frequent primary headaches is in the very period that encompasses occupationally and reproductively active years [28,29,30,31,32]. In a Dutch study with 1781 employees who received a questionnaire, of the 1082 who responded (60.8%), the prevalence of headaches was 53.3%, with 39.8% of participants having at least one type of headache [7]. We obtained similar results, i.e., 56.6% of all our subjects had headaches. Somewhat more favorable results were reported by Sato and colleagues: out of 721 workers with headaches, 307 (42.6%) reported experiencing at least one headache per month [33].

With regard to individual headache types, in the Dutch study, 12.4% of the responders had migraine headaches, 17% had tension-type headaches, and 10.4% another type of headache [7]. Similar prevalence was found in other studies [1,7,31,32]. In our study, 16.53% of subjects had migraines, 21.91% had tension-type headaches, and 18.19% had some other type of headache. Other types of headaches in our study were not limited to other primary headaches, but all other primary and secondary headaches not belonging to migraines and/or tension-type headaches. This probably explains the greater percentage of other-type-headache sufferers in our study.

Headaches are not only an individual problem for headache sufferers, but also a socioeconomic problem with substantial impact on patient daily-life activities, including work. The indirect cost of pain may be estimated with the number of missed work days [34]. Most of our subjects had never missed work regardless of headache type. The least absenteeism was found in subjects with tension-type headaches; 93.3% of these subjects had never missed work due to a headache. Occasional absenteeism was significantly more frequent in subjects with migraines (20.1%) compared to subjects with tension-type headaches (6.3%) and other headaches (7.5%). Only 1.8% of subjects with migraine always missed work during an attack. With regard to migraine subtypes, subjects with migraines with aura missed work twice as frequently as those with other migraine subtypes. With regard to absence from work in the past 30 days, subjects with migraines (7.1%) missed work statistically significantly more frequently compared with subjects with tension-type headaches (2.2%) and other headache types (2.2%). On average, 3.6% of our subjects had missed work in the past 30 days, which was a higher percentage than that reported in other studies [1,7]. In a study by Pop and associates, 2.5% of headache sufferers missed 1 or more work days in a month due to a headache [7]. In our study, subjects with migraines with aura and chronic tension-type headaches missed more work days compared to those with other headache subtypes, and none of the subjects with episodic tension-type headaches missed a work day in the past 30 days.

The reduced work performance of a person with a headache does not only depend on lost days or hours of work. Hours spent working with a headache are also important. Stewart and colleagues conducted a cross-sectional study of working adults with common pain conditions (arthritis, back pain, headaches, and other musculoskeletal issues) [35]. Headaches were the most common (5.4%) pain condition resulting in lost productive time [35]. In our study, subjects with migraines and tension-type headaches most frequently worked with a headache for 1–8 or 9–24 h per month in similar percentages, whereas in the group of other headaches, 49.5% of subjects worked in spite of a headache for 1–8 h.

Work efficiency while working with a headache was reported in the range of 66–90% in all three headache groups. This finding is comparable with that of the Dutch study, where subjects who continued working in spite of a headache reported a 20.7% loss of productivity [7]. Work efficiency while working with a headache was lower in subjects with migraines compared to that of subjects with tension- and other-type headaches. Regarding individual headache subtypes in our study, the least efficient while working with a headache were subjects with migraines with aura.

Numerous studies demonstrated that headaches adversely affect various aspects of the sufferer’s life [25,26,27,28,29,30]. Trying to understand how a headache influenced work efficiency in our subjects, we asked them how a headache affects their work by offering several answers and allowing them to specify some other effects of their headaches. Our results showed that the same answers dominated in all three headache groups. However, migraine subjects were the least efficient. More than two-thirds of subjects in all three headache groups reported that headaches did not cause them to work less attentively and/or precisely, or slower. On the other hand, more than two-thirds of subjects with headaches reported that they had to put more effort in their work. A small percentage of subjects reported other effects of headache on their work. Some studies confirmed that most of lost pain-related productive time occurs while employees are at work and is in the form of reduced performance [35]. Few studies confirmed that direct medical costs dominated [36].

A realistic, rational, and economical approach with adherence to the current guidelines for diagnosis and treatment is frequently almost an impossible task for a practicing physician. In less developed countries, such as Serbia, this problem is even greater. However, research showed that the better treatment of headache patients improves their quality of life, and their occupational and social functioning [37,38,39,40].

Periodic employee examinations should also include headaches. Earlier diagnosis and treatment would contribute to the greater work efficiency of patients. Moreover, migraineurs should be made aware that the early intervention and management of migraines are very important in order to gain the highest benefit from preventive treatment [41].

There are some limitations in our study. Given the large number of established types and subtypes of headaches in ICHD, we could not prepare a questionnaire to cover all of them. A limitation of the study is the impossibility of diagnosing secondary headaches, which requires appropriate diagnostics. Furthermore, the tested sample could be larger, and research could also be done in the category of headaches with lower prevalence.

## 5. Conclusions

Headaches significantly affect the work and work efficiency of headache sufferers through work absenteeism due to the headache and working in spite of the headache, which reduces productivity. Furthermore, loss was greater due to reduced efficiency than due to absenteeism. The least productive were subjects suffering from migraines with aura. All this indicated that headaches, and especially migraines, are of great medical and socioeconomic significance. Headaches, especially migraines, represent a burden for patients, their families, and society at large.

## Figures and Tables

**Table 1:** Number (*n*) and percentage (%) of sufferers who lost work days due to a headache in the past month. TTHs, tension-type headaches.

Lost Work Days	*n* and %	Migraines (*n* = 169)	TTHs (*n* = 224)	Other Headache Types (*n* = 186)	Overall Headaches (*n* = 579)
No	*n*	157	219	182	558
	%	92.9	97.8	97.8	96.4
Yes	*n*	12	5	4	21
	%	7.1 *	2.2	2.2	3.6

* Given answer significantly prevalent within row.

**Table 2:** Number (*n*) and percentage (%) of subjects working in spite of a headache in a month.

Time (h)	*n* and %	Migraines (*n* = 169)	TTHs (*n* = 224)	Other Headache Types (*n* = 186)
0	*n*	8	15	27
	%	4.7	6.7	14.5 *
1–8	*n*	54	90	92
	%	32.0	40.2	49.5 *
9–24	*n*	56	89	42
	%	33.1	39.7 *	22.6
25–40	*n*	25	9	8
	%	14.8 *	4.0	4.3
≥41	*n*	26	21	17
	%	15.4 *	9.4	9.1

* Given answer significantly prevalent within row.

**Table 3:** Coefficient of discrimination (CD) between studied headache types in relation to impact of headaches on work.

Headaches at Work	Migraines	TTHs	Other Headache Types	CD
Working with headache.	25–40 *	9–24 h *	0 h *	0.056
	≥41 *		1–8 h *	
Efficiency while working with headache.	0–40% *	66–90% *	91–100% *	0.042
	41–65% *			
Work absence during an attack.	Sometimes *	Never *	Always	0.018
	Always			
Effort.	More effort *	-	No effort *	0.011
Attention and precision.	Reduced *	Not reduced	Not reduced *	0.010
Work speed.	More slowly *	-	Same speed *	0.008
Other effects.	-	No other effects *	Other effects *	0.008
Work absence per month.	Yes *	No	Yes	0.000

* Given answer is significantly prevalent within the row.

**Table 4:** Self-reported efficiency of subjects, number (*n*) and percentage (%), while working with headaches from the studied headache types.

Efficiency	*n* and %	Migraines (*n* = 169)	TTHs (*n* = 224)	Other Headache Types (*n* = 186)
0–40%	*n*	30	11	16
	%	17.8 *	4.9	8.6
41–65%	*n*	52	47	34
	%	30.8 *	21.0	18.3
66–90%	*n*	78	141	94
	%	46.2	62.9 *	50.5
91–100%	*n*	9	25	42
	%	5.3	11.2	22.6 *

* Given answer is significantly prevalent within the row.

**Table 5:** Impact of headaches on work in relation to headache subtype. Cntr, contribution of item to characteristics; MWoA, migraine without aura; MWA, migraine with aura.

Headache Impact	MWoA	MWA	Infrequent TTHs	Frequent TTHs	Chronic TTHs	Cntr %
Working with headache.	-	25–40 h	0 h *	9–24 h *	25–40 h *	51.93
		≥41 h	1–8 h *		≥41 h *	
Efficiency while working with headache.	0–40% *	0–40%	66–90%	66–90% *	91–100% *	19.29
	41–65% *					
Work absence.	-	Sometimes *	Never *	-	Always *	12.76
					Sometimes	
Effort.	-	-	No effort *	-	More effort *	6.53
Attention and precision.	Reduced	Reduced *	-	-	Not reduced *	6.23
Work absence per month.	-	-	No *	-	yes *	3.26
Homogeneity %	53.40	54.55	71.55	66.67	77.78	

* Given answer significantly prevalent within row.

## Supplementary Materials

The following are available online at https://www.mdpi.com/1660-4601/17/18/6918/s1, Questionnaire S1: Questionnaire about the Characteristics of Headaches.
