# Supplementary material for: Impact of Individual Headache Types on the Work and Work Efficiency of Headache Sufferers
Source: Int J Environ Res Public Health. 2020 Sep 22;17(18):6918. doi: 10.3390/ijerph17186918 (PMC7560060; doi:10.3390/ijerph17186918)
Supplement: Supplementary file 1 [file ijerph-17-06918-s001.pdf]

International Journal of
Environmental Research and Public Health

Questionnaire about the Characteristics of Headaches

1. How old were you when the headache occurred for the first time? ________________

2. How often do you have headache attacks?
a) < once a month
b) 1 to 14 days per month
c) > 14 days per month
d) _________________

3. My headaches last:
a) Less then half an hour
b) 4 hours to 3 days
c) From half an hour to several days, 7 days the longest
d) More than 7 days

4. My headaches are located:
a) On one side of the head – with occasional change of side
b) Always on one side
c) On both sides
d) All around the head
e) _____________________

5. The intensity of the pain is: (from 1 to 10)
a) Mild (1-3)
b) Moderate (4-6)
c) Severe (7-10)
d) Insufferable

6. The quality of the pain you experience is:
a) Pulsating
b) Pressing or tightening
c) ______________________

7. Do your headaches worsen after physical activities such as walking or staircase climbing?
a) Yes b) No

8. Do you avoid routine physical activities because you are afraid they might trigger your headache?
a) Yes b) No

9. Are the headaches accompanied by?
a) Nausea
1) Yes
2) No
b) Vomiting
1) Yes
2) No
c) Photophobia (light sensitivity)
1) Yes
2) No
d) Phonophobia (noise sensitivity)

10. Do you have temporary visual, sensory or speech disturbance?
a) Yes b) No

11. Do you, during a headache attack, have tension and/or heightened tenderness of head or neck muscles?
a) Yes b) No

12. Do you have any body numbness or weakness?
a) Yes b) No

13. Do you have any indications of oncoming headache?
a) Yes, ________________________
b) No, none

14. Headache is usually triggered by:
a) Certain foods, most commonly ___________________
b) Certain drinks, most commonly ___________________
c) Stress
d) Fatigue
e) Strong odors
f) Changes in atmospheric pressure
g) Menstrual periods
h) _____________________

15. In the half or my visual field, lasting 5 minutes to an hour, along with the headache attack or an hour before one, there are following symptoms:
a) Blinking lights, spots or lines
b) Loss of sight
c) No symptoms

16. Along with the headache attack or an hour before one I have following sensory symptoms, lasting 5 minutes up to an hour:
a) A prickling sensation
b) Numbness
c) No symptoms

17. Headache usually occurs (only for female respondents):
a) 3 to 7 days before menstrual period
b) 2 days before and 3 days during the menstrual period
c) Unrelated to menstrual period
d) ____________________________

18. Headache usually occurs (only for female respondents):
a) Only during menstrual period
b) During menstrual period but at other times as well
c) Unrelated to menstrual period
